# Quantitative computed tomography body composition analysis for risk stratification in bronchiectasis

**DOI:** 10.1186/s12931-026-03670-x

**Published:** 2026-04-17

**Authors:** Umberto Semenzato, Virginia Santello, Giulia Fichera, Daniele Previtero, Chiara Contin, Andrea Rastelli, Rossella Valvason, Marta Zuffellato, Anna Ferrari, Alessandro Micelli, Chiara Giraudo, Andrea Sattin, Annamaria Cattelan, Paolo Spagnolo, Mariaenrica Tinè

**Affiliations:** 1 Respiratory Medicine, Department of Cardiac, Thoracic, Vascular Sciences and Public Health, University of Padova, Padua, Italy; 2 Unit of Advanced Clinical and Translational Imaging, Department of Cardiac, Vascular Sciences and Public Health, Padova University, Thoracic, Padova, Italy; 3 Respiratory Disease Unit, Ospedale dell’Angelo, Mestre, Venice, Italy; 4 Department of Pneumology, Cittadella Hospital (PD), ULSS6 Euganea, Cittadella, Italy; 5 Pulmonology Unit, Dolo-Mirano Hospital, Serenissima, Venice, AULSS3 Italy; 6 Pulmonology Unit, San Bassiano Hospital, Bassano Del Grappa, Italy; 7 Infectious and Tropical Diseases Unit, Padova University Hospital, Padua, Italy

**Keywords:** Bronchiectasis, Sarcopenia, Myosteatosis, Personalized medicine, Nutritional status, Computed tomography, Comorbidities, Nontuberculous mycobacteria

## Abstract

**Background:**

Nutritional status and sarcopenia are established contributors to morbidity and mortality in chronic respiratory diseases. This study aimed to assess the prevalence and impact of body composition features extracted from computed tomography (CT) in patients with non-CF bronchiectasis.

**Methods:**

We analysed data from 112 patients. Muscle and subcutaneous fat area and density were assessed by semi-automatic segmentation at the level of the 12th thoracic vertebra using the mediastinal window of routine chest CT scans. Clinical and functional features were compared between patients with and without body composition abnormalities. Associations between CT-derived body composition parameters and clinical outcomes were explored.

**Results:**

The majority of patients were female (86/112, 77%) and the median age was 66 years (56–74). Myosteatosis (low muscle density) affected 75 of 112 (66%) patients. Patients with myosteatosis (MSp) were older than those without (wMSp), median age 70 (61–77) vs. 58 (45–69) years (*p* < 0.001), and had lower FEV_1_, 81 (69–95) vs. 89 (78–101) % pred. (*p* = 0.038) and MEF_50_, 48 (25–66) vs. 61 (44–74) % pred. (*p* = 0.043). The incidence of non-tuberculous mycobacteria (NTM) pulmonary disease was higher in MSp compared to wMSp (19% vs. 3%; *p* = 0.043). Muscular density negatively correlated with the Bronchiectasis Severity Index (*r* = − 0.32; *p* = 0.012), FACED (*r* = − 0.38; *p* = 0.03) and E-FACED (*r* = − 0.35; *p* = 0.007) scores. Myosteatosis was not related to gender, radiological extension of bronchiectasis, bacterial colonization or laboratory data.

**Conclusions:**

Myosteatosis is highly prevalent in patients with bronchiectasis and is associated with worse airflow obstruction, higher disease severity, and increased frequency of NTM pulmonary disease, independently from BMI. Abnormal body composition as defined by CT assessment may provide clinically relevant information beyond traditional anthropometrics and complement existing severity scores for risk stratification in patients with bronchiectasis.

**Supplementary Information:**

The online version contains supplementary material available at 10.1186/s12931-026-03670-x.

## Background

Non-cystic fibrosis bronchiectasis (hereafter called bronchiectasis) is a chronic respiratory disease defined by a clinical syndrome characterized by cough, sputum production, and recurrent bronchial infections, along with radiological evidence of abnormal and permanent dilation of the bronchi [1, 2].

Historically, bronchiectasis has been a neglected disease. Its overall prevalence ranges from 53 to 566 cases per 100,000, varying across different countries and cohorts, with higher rates observed in older adults and females [2, 3]. Despite this growing awareness, underestimation of bronchiectasis remains prevalent among healthcare professionals across various settings [4], and a diagnostic delay, even if improved compared to the past decades, still exists.

In patients with suspected bronchiectasis, high-resolution computed tomography (CT) is required to confirm the diagnosis and to characterize disease extent and morphology [5]. Once diagnosed, a standardized etiological work-up is recommended, given the marked heterogeneity of underlying causes and associated conditions [1, 5].

The goals of treatment include exacerbation prevention, reducing symptoms, improving quality of life and stopping disease progression. Bronchiectasis, in fact, may lead to substantial morbidity and mortality despite optimal management [2]. A key challenge in disease management lies in the identification of patients at high risk of poor outcomes, and for this purpose, several clinical scoring systems have been developed through the years. The Bronchiectasis Severity Index (BSI) combines age, body mass index (BMI), FEV_1_, previous hospitalization, exacerbation frequency, colonization status and radiological appearances. The score was designed to predict future exacerbations and hospitalizations, health status, and mortality over 4 years [6]. FACED (FEV_1_, Age, chronic Colonization, Extension and Dyspnoea) is another tool used to predict 5-year mortality in patients with bronchiectasis [7]. Finally, Bronchiectasis Aetiology Comorbidity Index (BACI) considers comorbidities of patients and may serve as a predictor of 5-year mortality, hospitalizations, exacerbations and health-related quality of life [8].

Among the comorbidities that significantly impact on bronchiectasis, the association with nontuberculous mycobacterial (NTM) pulmonary disease is not only common but also clinically relevant, as these infections are associated with a more severe disease course, increased morbidity, and higher healthcare utilization [9]. NTM pulmonary disease is often associated with female sex and lower BMI [10].

Of note, in both bronchiectasis and nontuberculous mycobacterial (NTM) pulmonary disease, nutritional aspects play a significant role. Indeed, body mass index (BMI) lower than 18.5 kg·m⁻² is both a risk factor for the development of NTM pulmonary disease [11] and a predictor of mortality in Bronchiectasis Severity Index [6]. Moreover, recent evidence indicates that malnutrition, low BMI, and sarcopenia are associated with poor respiratory function, increased risk of hospitalization, and higher mortality in patients with bronchiectasis [12]. Further underlying the importance of body composition in these patients, subjects with bronchiectasis show significant reduction in muscle strength and endurance compared to controls, impairment associated with worse dyspnoea, health related quality of life and functional capacity over one year [13].

Even though the role of BMI is well established in many chronic respiratory diseases such as Chronic Obstructive Pulmonary Disease (COPD) [14], it does not fully reflect a patient’s nutritional status or body composition. Indeed, muscle function and quality, which are not captured by BMI, also impact on patient’s outcomes. Muscle changes, including sarcopenia and myosteatosis, have emerged as a significant factor in chronic respiratory diseases, mainly in COPD [15].

Sarcopenia is a progressive and generalized skeletal muscle disorder characterized by low muscle strength and quality or quantity, and low physical performance [16]. Myosteatosis, which is linked to sarcopenia, refers to pathological fat infiltration into muscle tissue [17]. According to the international working groups, Dual-energy X-ray absorptiometry (DXA) is the main tool to assess sarcopenia [18]. Nevertheless, other radiological techniques like ultrasound, CT and magnetic resonance imaging can be applied for the evaluation of body composition [19] by providing both qualitative and quantitative assessments of skeletal muscle. In particular, when used to evaluate body composition, especially muscle mass and quality, CT may provide prognostic value in various diseases [20–22]. This approach was initially used in oncological patients, but recent data has demonstrated its utility in chronic conditions, including COPD [21, 22] and other systemic diseases. For example, among patients with systemic sclerosis, 72% exhibit myosteatosis as assessed by HRCT, which is associated with an increased risk of mortality [23].

Only recently has attention shifted toward peripheral skeletal muscle in bronchiectasis. A small retrospective study in patients hospitalized for an exacerbation suggested that CT-derived pectoralis muscle area is an independent predictor of mortality. While recent evidence has highlighted the potential role of imaging-derived sarcopenia markers in bronchiectasis [24], no study has yet provided a comprehensive evaluation of body composition encompassing both muscle mass and muscle quality, nor has fully explored its prognostic impact in clinically stable patients.

Despite the increasing use of imaging techniques for the assessment of body composition, data on a comprehensive imaging-based evaluation of both muscle mass and muscle quality in patients with bronchiectasis remains limited, particularly in clinically stable patients. Therefore, the present study explores an innovative approach by leveraging routinely performed thoracic HRCT scans to assess both muscle quantity and quality in patients with bronchiectasis, aiming to determine their prevalence and to evaluate their prognostic impact as well as their associations with comorbidities.

## Methods

### Study population

This retrospective observational study included 112 consecutive patients with bronchiectasis not due to cystic fibrosis referred to our Bronchiectasis outpatient clinic at the Respiratory Disease Unit of Padua University Hospital (Padua, Italy) from February 2020 to December 2023.

All patients had a diagnosis of bronchiectasis confirmed by a pulmonologist experienced in the disease and a thoracic radiologist. Inclusion criteria were: age ≥ 18 years, a clinical history characterized by current or previous history of cough, chronic sputum production, or recurrent respiratory infections; and chest CT evidence of bronchiectasis (bronchial dilatation) affecting one or more lobes. Exclusion criteria included a diagnosis of cystic fibrosis, traction bronchiectasis associated with interstitial lung disease, inability to provide informed consent or denial of consent.

Baseline data included age, sex, smoking history, occupational exposure, body mass index (BMI), exacerbation history and allergies. Exacerbation was defined as a deterioration in three or more of the following key symptoms for at least 48 h (cough, sputum volume and/or consistency, sputum purulence, breathlessness and/or exercise tolerance, fatigue and/or malaise, and hemoptysis) combined with a clinician’s judgment that a change in bronchiectasis treatment was required [25].

According to the British Thoracic Society guidelines [5], the etiology of bronchiectasis was determined and baseline blood tests were collected. We also gathered information about comorbidities and daily symptoms.

All patients underwent comprehensive pulmonary function tests at baseline, with spirometry and diffusing capacity of the lung for carbon monoxide (DLCO).

Radiological characteristics of bronchiectasis were evaluated, specifically bronchiectasis type (cylindrical, varicose, and cystic) and the number of affected lobes (considering the lingular segment as a separate lobe) based on chest CT scans.

Microbiological data were recorded, specifically culture results for bacteria, mycobacteria, and fungi from sputum, bronchoaspirate and/or bronchoalveolar lavage; galactomannan antigen on bronchoalveolar lavage was also searched for when clinically indicated. Chronic airway colonization was defined as the presence of potentially pathogenic bacteria in sputum (or other respiratory samples) culture on two or more occasions at least 3 months apart within a year [26]. NTM pulmonary disease was diagnosed according to current guidelines [10].

Based on the above data, for each patient the following severity scores were calculated: BSI, FACED, E-FACED, and BACI [6–8, 27, 28].

The study conformed to the Declaration of Helsinki and was approved by the local Institutional Review Board (approval no. 6191/AO/25); patients provided informed consent. Clinical trial number: not applicable.

### Radiological evaluation of muscle density and myosteatosis

**Fig. 1:**
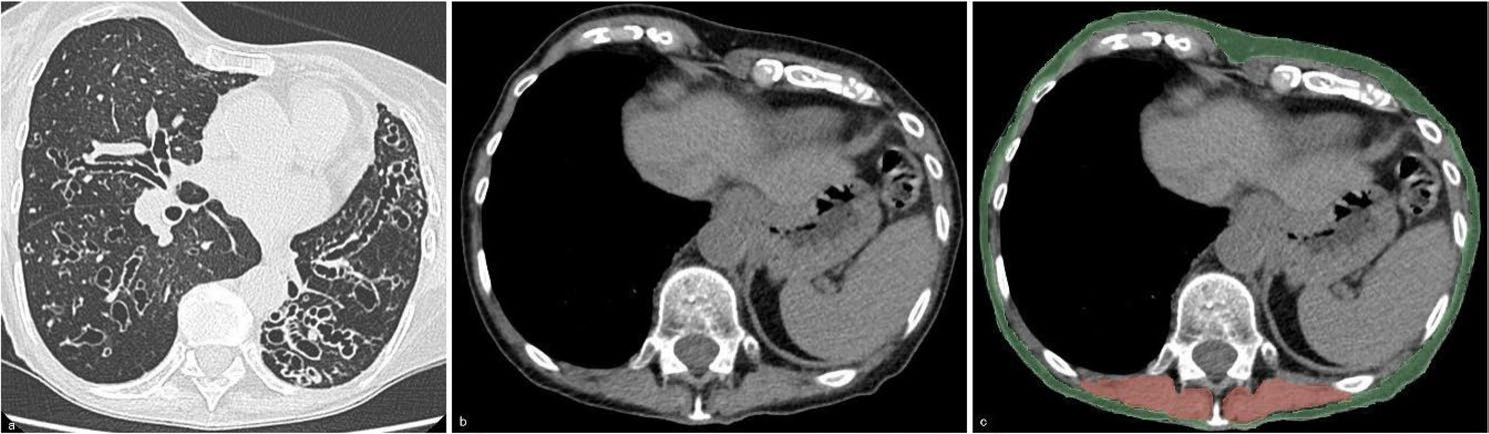
Axial chest CT of a 59-year-old female patient affected by severe bilateral bronchiectasis **A**. In (**B**), the soft tissue window at the 12th thoracic vertebra level which has been used for the segmentation. In (**C**), the segmentation of the subcutaneous tissue (green area) and paravertebral muscle (red area)

Using the open-source software 3D Slicer [29, 30], for each patient a semi-automatic segmentation of the paravertebral muscles and subcutaneous tissue was performed at the level of the 12th thoracic vertebra using the soft tissue window and applying standardized thresholds: −29 to 150 Hounsfield Unit (HU) for muscles and − 190 to − 30 HU, for fat). Then muscle and subcutaneous area and density (i.e. HU) were extracted (Fig. 1) [23]. In addition, the vertebral muscle index (VMI) was calculated as the muscle cross-sectional area normalized for height squared (cm²/m²) to provide an index of muscle quantity. A threshold value of 30 Hounsfield Units was used to define low muscle density and myosteatosis, as previously reported [31]. If patients underwent multiple chest CT scans, the scan performed closest in time to the baseline clinical and functional assessment was selected for analysis, in order to ensure temporal alignment between imaging-derived body composition parameters and clinical variables. The choice of the T12 vertebral level, which is included in routine chest CT scans, has been validated as an alternative to the traditional L3 level in scenarios where abdominal imaging is not available [20].

### Statistical analysis

Continuous variables were expressed as median and interquartile range (IQR), while categorical variables were presented as counts and percentages. Comparisons among groups were evaluated using the Kruskal-Wallis and Mann-Whitney U tests. Categorical variable distributions were compared using the χ² test. Spearman’s rank correlation coefficient was used to evaluate the relationship between different continuous variables. All statistical analyses were performed with IBM SPSS Statistics (IBM Corp. Released 2023. Version 29.0.2.0 for Windows, Armonk, NY: IBM Corp) and GraphPad Prism (version 10.0.0 for Windows, GraphPad Software, Boston, Massachusetts USA); a p-value < 0.05 was considered statistically significant.

## Results

### Demographic and clinical characteristics

Overall, 112 patients were enrolled in the study. The majority were female (86/112, 77%) with a median age of 66 years (IQR 56–74). The etiology of bronchiectasis is detailed in Figure S1.

Patients were divided into two groups based on muscle density: those with myosteatosis (MSp) (*n* = 75; 67%) and those without (wMSp) (*n* = 37; 33%). MSp were older than wMSp, while BMI did not differ between the two groups (Table 1). Other baseline characteristics were comparable between the two groups, with no significant differences in smoking history, exacerbation in the past year or gender distribution.

**Table 1:** Demographic characteristics of the study population stratified by myosteatosis

Variable	Total population (*n* = 112)	MSp (*n* = 75)	wMSp (*n* = 37)	*p*-value
Age (years)	66.17 (55.51–73.83)	70.42 (60.75–76.53)	57.58 (44.96–69.15)	< 0.001
Male, n (%)	26 (23%)	14 (19%)	12 (32%)	0.085
Pack-years	0 (0–4)	0 (0–9.25.25)	0 (0–3)	0.903
Occupational exposure, n (%)	25 (22%)	14 (19%)	11 (30%)	0.211
BMI (kg·m⁻²)	21.92 (19.74–24.85)	22.05 (20.08–25.06)	21.63 (19.07–23.40)	0.146
Subjects with BMI < 18.5 n (%)	11 (10%)	5 (6%)	6 (16%)	0.106
mMRC Dyspnea Scale	1 (0–1)	1 (0–1)	0 (0–1)	0.300
Exacerbations in the previous year	1 (0–2)	1 (0–2)	1 (0–2.75.75)	0.775
Cylindrical bronchiectasis	83 (74%)	58 (77%)	25 (68%)	0.177
Varicose bronchiectasis	21 (19%)	14 (19%)	7 (19%)	
Cystic Bronchiectasis	8 (7%)	3 (4%)	5 (14%)	
Number of lobes involved	3 (2–4)	3 (2–4)	3 (2–4)	0.820
Bronchiectasis Severity Index (BSI)	5 (3–8)	6 (3–8)	5 (2.75–8.75)	0.280
FACED score	1.5 (1–3)	2 (1–3.5.5)	1 (1–2)	0.126
E-FACED score	3 (1–5)	3 (1–5)	2 (1.75–3.75)	0.240
BACI	0 (0–3)	0 (0–3)	0 (0–3)	0.468
Bacterial colonization	29 (26%)	20 (27%)	9 (24%)	0.790
Long-term macrolides	24 (29%)	19 (35%)	5 (17%)	0.069

Data are expressed as median (interquartile range) or number (percentage)

*Abbreviations*: *MSp * Patients with myosteatosis, *wMSp * Patients without myosteatosis, *BACI * Bronchiectasis Aetiology Comorbidity Index, *BMI * Body Mass Index, *E-FACED * Exacerbations–FACED score, *FACED * Forced Expiratory Volume in 1 second, Age, Chronic colonization, Extension, Dyspnoea score, *mMRC * Modified Medical Research Council

BMI showed a weak inverse correlation with muscle density (*r* = − 0.202, *p* = 0.033). Muscle area moderately correlated with body weight (*r* = 0.55; *p* < 0.001) and BMI (*r* = 0.45; *p* < 0.001), whereas its correlation with height was weak (*r* = 0.31; *p* < 0.001).

Subcutaneous fat attenuation (density) inversely correlated with BMI (*r* = − 0.56, *p* < 0.001) and body weight (*r* = − 0.37, *p* < 0.001). In contrast, subcutaneous fat cross-sectional area showed a strong positive correlation with body weight (*r* = 0.60, *p* < 0.001) and BMI (*r* = 0.79, *p* < 0.001).

VMI showed a moderate correlation with BMI but was not associated with muscle density or with any clinical or functional parameters (see Supplementary Results).

### Pulmonary function tests

**Fig. 2:**
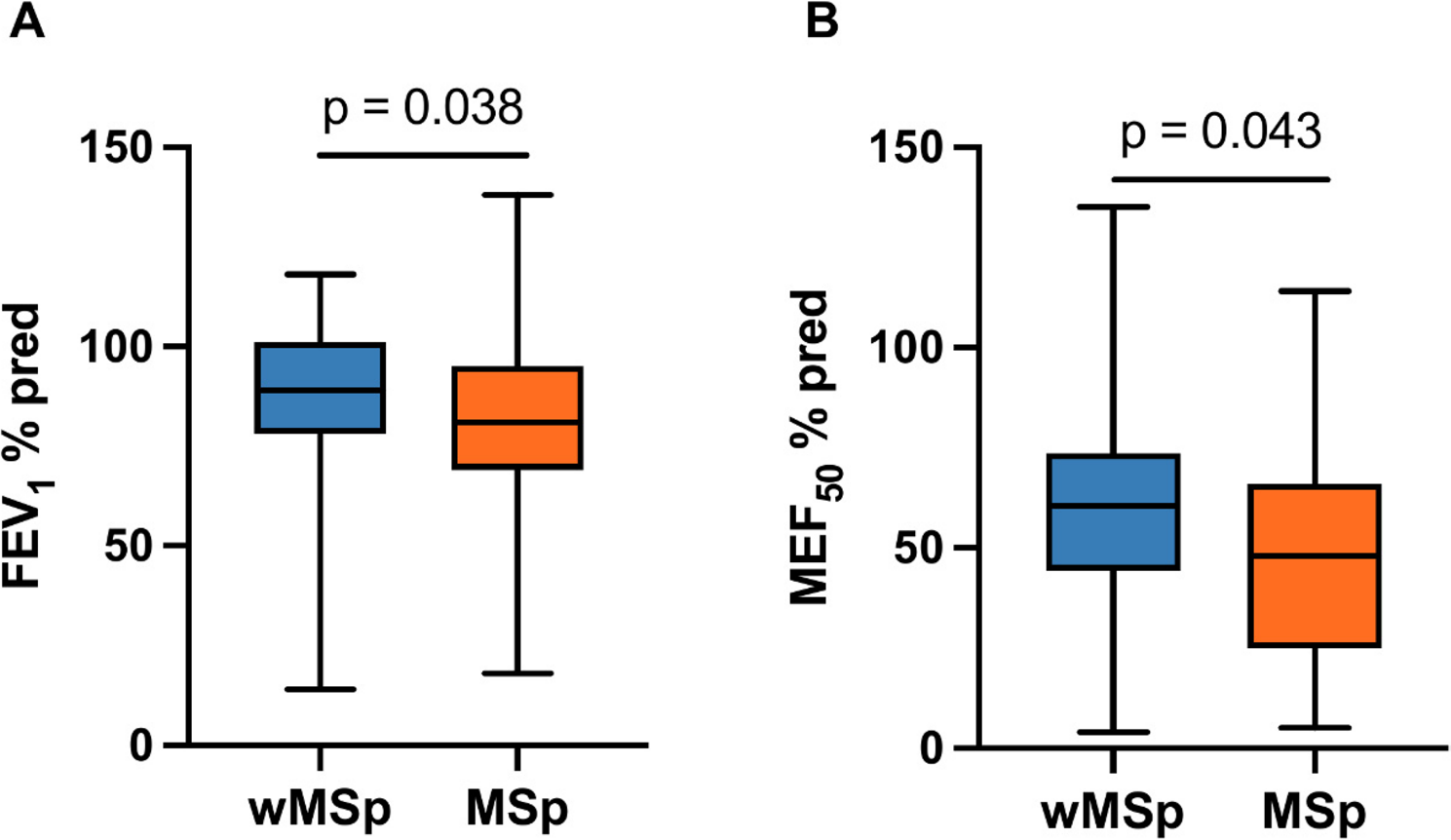
**A** FEV₁ % predicted in patients with myosteatosis (MSp) versus without (wMSp); (**B**) MEF₅₀ % predicted in MSp versus wMSp. Both airflow parameters were significantly lower in the myosteatosis group

Pulmonary function was assessed using global spirometry and DLCO. Patients with myosteatosis had significantly reduced expiratory airflow compared to wMSp. In particular, FEV₁ and mid-expiratory flow (MEF₅₀) were both significantly lower in the MSp group, in terms of both absolute (liters) and percent predicted values (Table 2; Fig. 2). Forced vital capacity (FVC), peak expiratory flow (PEF) and total lung capacity (TLC) were also lower in MSp, but only when expressed as absolute values (Table 2). The FEV₁/FVC ratio was slightly lower in MSp, but without reaching statistical significance (*p* = 0.089). DLCO did not differ among the two groups.

**Table 2:** Pulmonary function data for the study population

Variable	Total population (*n* = 112)	MSp (*n* = 75)	wMSp (*n* = 37)	*p*-value
FEV₁ (L)	2.05 (1.52–2.40)	1.88 (1.44–2.22)	2.31 (2.03–2.95)	< 0.001
FEV₁ (% pred.)	84 (73.5–96.25.5.25)	81 (69–95)	89.11 (78–101)	0.038
FVC (L)	2.83 (2.21–3.39)	2.58 (2.08–3.14)	3.28 (2.84–3.90)	< 0.001
FVC (% pred.)	96 (84.75–109.25.75.25)	94 (82–107)	99 (85.5–111)	0.187
FEV₁/FVC	0.73 (0.66–0.78)	0.72 (0.63–0.78)	0.75 (0.69–0.80)	0.089
PEF (L)	5.28 (4.15–6.83)	4.90 (3.85–6.07)	6.22 (5.04–7.16)	0.004
PEF (% pred.)	86 (70–101)	85 (70–100)	90 (75.25–104.50)	0.230
MEF_50_ (L)	1.83 (1.25–2.83)	1.70 (0.92–2.31)	2.33 (1.64–3.07)	0.009
MEF_50_ (% pred.)	51 (33–71)	48 (25–66)	60.5 (44.25–73.50)	0.043
TLC (L)	4.55 (3.94–5.16)	4.38 (3.72–5.02)	5.02 (4.18–5.54)	0.016
TLC (% pred.)	86 (78–95)	85 (78–94)	92 (78.5–100.5.5.5)	0.317
RV (L)	1.97 (1.66–2.28)	1.98 (1.62–2.25)	1.95 (1.68–2.41)	0.801
RV (% pred.)	97 (83.5–113.25.5.25)	93 (81.5–112.5.5.5)	99 (86.5–117)	0.392
RV/TLC	0.43 (0.39–0.48)	0.44 (0.41–0.49)	0.40 (0.38–0.45)	0.431
DLCO (% pred.)	72.5 (65–83)	71 (64–83)	75 (66–85)	0.313

Data are expressed as median (interquartile range)

*Abbreviations*: *MSp * Patients with myosteatosis, *wMSp * Patients without myosteatosis, *FEV*_1_ Forced Expiratory Volume in 1 s, *FVC* Forced Vital Capacity, *PEF * Peak Expiratory Flow, *MEF*_50_ Maximal Expiratory Flow at 50% of FVC, *TLC* Total Lung Capacity, *RV* Residual Volume, *DLCO * Diffusing capacity of the lungs for carbon monoxide, *pred*. predicted

Across the entire study population, thoracic muscle density (in HU) correlated significantly with multiple pulmonary function parameters. Notably, higher muscle density was associated with better airflow and lung volumes; indeed, we observed moderate positive correlations between muscle density and FEV_1_ (*r* = 0.46, *p* < 0.001, Figure S2), FVC (*r* = 0.47, *p* < 0.001) and MEF_50_ (*r* = 0.403, *p* < 0.001) absolute values. Correlations with % predicted values were weaker but still positive for FEV₁% (*r* = 0.23, *p* = 0.016), FVC% (*r* = 0.19, *p* = 0.05), PEF% (*r* = 0.27, *p* = 0.006), and MEF₅₀% (*r* = 0.32, *p* < 0.01). Muscle density also showed a weak positive correlation with FEV₁/FVC (*r* = 0.27, *p* = 0.005) and TLC (*r* = 0.36, *p* < 0.001; *r* = 0.23 for TLC% predicted, *p* = 0.02). There was no correlation between muscle density and DLCO. Fat density showed a weak positive correlation with TLC (*r* = 0.20, *p* = 0.03) and RV (*r* = 0.26, *p* = 0.007).

### Bronchiectasis severity scores

Bronchiectasis severity was evaluated using the BSI, BACI, FACED and E-FACED scores. They did not differ significantly between MSp and wMSp groups (Table 1). However, across the entire cohort, muscle density showed a significant inverse correlation with BSI (*r* = − 0.32, *p* = 0.012, Figure S3), FACED (*r* = − 0.38, *p* = 0.003) and E-FACED (*r* = − 0.35, *p* = 0.007), suggesting that poorer muscle quality is associated with more severe disease. There was a trend towards a negative correlation between muscle density and BACI (*r* = − 0.249, *p* = 0.062). Muscle area, fat area or fat density showed no correlation with any disease severity scores.

### Serological parameters

Fat density showed moderate positive correlations with serum IgM (*r* = 0.3; *p* = 0.03), IgG (*r* = 0.479; *p* = 0.002), IgA (*r* = 0.339; *p* = 0.029) and alpha-1 antitrypsin levels (*r* = 0.53; *p* = 0.007). No further significant relationships were observed between muscle or fat measurements and other serological parameters; similarly, muscle density was not significantly correlated with immunoglobulin levels.

### Microbiologic isolation

There was no significant difference either in the overall rate of positive respiratory cultures between MSp and wMSp, or in the proportion of patients meeting the definition of chronic bacterial colonization (Table 1).

However, NTM pulmonary disease was diagnosed in 10 patients (13%) in the MSp group, compared to only 1 patient (3%) in the wMSp group (*p* = 0.043).

No other significant differences in chronic bacterial colonization were observed between the two groups.

## Discussion

This is the first study to evaluate the prevalence and impact of body composition metrics extracted from HRCT on patients with bronchiectasis. To date, studies in bronchiectasis have mainly focused on traditional anthropometric parameters, such as BMI [6], while assessment of sarcopenia and muscle quality in this setting is lacking. Only recently has interest begun to emerge; Seo et al. examined pectoralis muscle area in hospitalized patients and found it to be predictive of mortality [32], whereas a pilot study of 20 patients with stable bronchiectasis confirmed the presence of sarcopenia in these patients, using ultrasound and magnetic resonance imaging [24].

Our study addresses this gap by providing systematic data in a larger cohort of clinically stable patients and by adopting a CT-based approach that can be readily integrated into routine clinical practice. A major finding of the study was that myosteatosis, a key component of malnutrition and a risk factor for poor clinical outcomes, was highly prevalent (66%) in our cohort. Considering the low prevalence of sarcopenia, which is seldom observed in healthy subjects (2–4%) [33], this high rate of muscle quality impairment aligns with the growing evidence linking body composition to chronic respiratory diseases, particularly COPD [34–36]. Indeed, recent studies report prevalence rates for low muscle density in COPD as high as 63%, with the highest rates observed in frail, institutionalized patients [37]. Our data indicates that bronchiectasis is associated with a degree of systemic muscle involvement comparable to that observed in advanced stages of other chronic diseases, highlighting the need for early recognition of this underappreciated condition [12]. Although statistically significant, the correlations between BMI and CT-derived body composition parameters were weak, showing that BMI is a poor marker of muscle–fat balance in patients with bronchiectasis.

With regard to myosteatosis, we observed a significant association between reduced muscle density and function impairment. Of note, patients with reduced muscle density (and therefore myosteatosis) exhibited significantly lower percent predicted value of FEV_1_ and MEF_50_ values compared to wMSp, while total lung capacity and DLCO were relatively preserved, suggesting a somewhat selective impact on airflow limitation. Other respiratory function parameters such as TLC, PEF and FVC were decreased in MSp only when measured as absolute value, not as percent predicted. Such results, contrary to FEV_1_ and MEF_50_, could be related to age, height or BMI. It is reasonable that subjects with myosteatosis have expiratory muscle weakness being unable to effectively empty their lungs. Nonetheless, RV and RV/TLC were slightly but homogeneously increased in patients with and without myosteatosis. A case could be made that a statistically significant difference in air trapping markers could be reached by expanding the study population. Moreover, impairment of expiratory muscle function may result in ineffective cough and impaired airway clearance, potentially contributing to an increased risk of NTM pulmonary disease. Of note, such evidence is consistent with previous observations in patients with COPD [38] where sarcopenia contributes to increased morbidity and mortality [38–40]. Similarly to COPD, the clinical course of bronchiectasis is punctuated by acute exacerbations that critically impact on prognosis [1, 2]. Seo et al. recently found that CT-derived pectoralis muscle area was the strongest independent predictor of mortality in patients hospitalized for bronchiectasis exacerbation, outperforming canonical risk factors such as diabetes, history of previous exacerbations and age [32]. In addition, low FEV_1_ and concomitant COPD, which characterize the frequent-exacerbator phenotype among bronchiectasis patients [41], might be exacerbated by the presence of sarcopenia. Indeed, COPD and bronchiectasis share many triggers of muscle wasting, such as physical inactivity, chronic hypoxemia, malnutrition, and systemic inflammation [24, 42, 43].

Moreover, as the paravertebral muscle density decreased, the values of the widely used multidimensional severity scores—BSI, FACED, and E-FACED—increased in our cohort. The correlation between muscle deterioration and BSI, which includes BMI among its variables, may not be surprising. Nonetheless, we did not observe a significant relationship between BMI and myosteatosis, challenging the reliance on BMI as a marker of nutritional status. While quantitative CT-based indices such as muscle area or vertebral muscle index primarily reflect muscle quantity, increasing evidence indicates that muscle radiodensity expressed in Hounsfield units provides more biologically meaningful information on muscle quality and fat infiltration, capturing heterogeneous and functionally relevant muscle alterations that are not detected by purely quantitative measures [17, 44]. Consistently, in our cohort VMI was not associated with clinical or functional parameters, whereas muscle density correlated with lung function and disease severity. Our findings are in line with the emerging research questioning the use of BMI in chronic diseases like COPD, where sarcopenia and muscle dysfunction can occur even in patients with normal or increased BMI [45–47]. The negative correlation between muscle density and BMI observed in our study, although weak, further suggests the need to complement BMI with more specific assessment tools of muscle health. Beyond muscle composition, myosteatosis may also reflect systemic vulnerability and frailty in patients with bronchiectasis. Frailty in bronchiectasis is increasingly recognised as a multidimensional condition characterised by malnutrition, chronic inflammation and reduced physical activity, and is associated with worse clinical outcomes independently of BMI. In this context, CT-derived muscle density may provide an imaging marker of early muscle dysfunction and systemic impairment, particularly when direct measurements of muscle strength or physical performance are not available.

Interestingly, in our study, subcutaneous adipose tissue density (a surrogate marker of lipid infiltration) showed a positive correlation with serum immunoglobulin (IgM, IgG, and IgA) and alpha-1 antitrypsin levels. This observation is novel and should be interpreted cautiously, as the cross-sectional design of the study does not allow conclusions regarding causality. Adipose tissue is increasingly recognised as an active endocrine organ that secretes a variety of cytokines and other bioactive mediators involved in immune and inflammatory pathways. In particular, subcutaneous adipose tissue may play a role in immune–metabolic crosstalk due to its susceptibility to immune cell infiltration [48]. We therefore speculate that alterations in subcutaneous adipose tissue composition may reflect broader systemic inflammatory or metabolic pathways. Similar associations between adipose tissue alterations and muscle dysfunction have been described in other chronic diseases, including systemic sclerosis and cancer [23, 49]. However, the clinical relevance and mechanistic basis of these findings in bronchiectasis remain to be clarified and warrant further investigation.

One of the most intriguing results of our study is the novel link between abnormal body composition and NTM pulmonary disease. The probability of having an NTM pulmonary disease was markedly higher in MSp compared to wMSp. While the association between bronchiectasis and NTM pulmonary disease is well established, particularly in patients with lower BMI or impaired immunity [9, 50], the relationship between sarcopenia and NTM has not been described previously. Our findings are further supported by a recent study by Hong et al. [51], which reported that CT-measured pectoralis muscle area was associated with clinical outcomes in patients with NTM pulmonary disease. Although that study evaluated muscle quantity rather than muscle density and did not specifically focus on bronchiectasis, together these observations suggest that CT-derived skeletal muscle alterations—both quantitative and qualitative—may reflect systemic vulnerability in patients with NTM-associated lung disease. Our novel finding gives rise to two hypotheses. The first is that muscle loss (myosteatosis) may be a marker of increased susceptibility to NTM infection in bronchiectasis, potentially due to the association between myosteatosis and systemic inflammation, immunosenescence, and reduced physical resilience [52]. Notably, patients predisposed to NTM lung disease often exhibit a slender body habitus and distinct immune–endocrine profiles (e.g., low leptin levels) suggestive of a host frailty that could facilitate infection [53]. The second possible interpretation is that NTM pulmonary disease may lead to muscle deterioration and chronic inflammation through the increased catabolic burden of infection, thereby causing or worsening myosteatosis. Although the first hypothesis seems more plausible, the retrospective nature of this study limits our ability to draw definitive conclusions, and future studies are required to clarify a cause–effect relation between sarcopenia and NTM susceptibility/presence. Our findings also suggest that addressing myosteatosis might be important in these patients; it raises the question of whether interventions aimed at improving muscle mass and quality (e.g., nutritional support or pulmonary rehabilitation incorporating exercise training) could potentially mitigate NTM infection risk and improve treatment outcomes. In support of this notion, recent studies have shown that hypercaloric, high-protein nutritional strategies and resistance exercise improve muscle mass, immune function, and quality of life in patients with bronchiectasis, particularly when integrated into multidisciplinary care programs [12].

There are limitations to our study that should be acknowledged. First, this is a single-centre study with relatively small sample size, which may limit the generalizability of our findings. The higher age observed in patients with myosteatosis may represent a potential confounder. However, age did not differ between patients with and without NTM pulmonary disease, and it is already incorporated within composite severity indices such as the BSI, thus reducing the likelihood that age alone explains the observed associations. Additionally, for the purpose of the study myosteatosis was defined according to a previously defined cut-off value for paravertebral muscle mass that has not been optimized in bronchiectasis, which could affect the generalizability of our results. Further studies should explore and establish optimal muscle density cut-offs for patients with bronchiectasis. We also acknowledge that data on muscle strength were not collected and that we did not perform additional assessments of muscle quantity (such as dual-energy X-ray absorptiometry or bioimpedance) to formally diagnose sarcopenia; nonetheless, CT scan is considered a reliable tool for non-invasive assessment of muscle quantity/mass [16]. In our study, we used CT to measure chest wall muscle density at the level of the 12th thoracic vertebra (T12). Even though the 3rd lumbar vertebra (L3) is the most commonly used reference point in the literature for assessing myosteatosis, measuring muscle mass at the T12 level has emerged as a practical and reliable alternative, particularly when lumbar images are unavailable or in case chest CT was performed for clinical reasons, as in our case [20, 31, 33]. Moreover, the retrospective nature of the study limits our ability to establish causal relationships between myosteatosis and clinical outcomes in bronchiectasis patients. In addition, longitudinal outcome data (including follow-up exacerbations, hospitalizations and mortality) were not systematically available, precluding direct evaluation of the prognostic impact of CT-derived body composition parameters. We also acknowledge that functional performance measures such as the 6-minute walk test or muscle strength assessments were not available in this cohort. Therefore, lung function alone cannot fully capture functional impairment. Information on lifetime occupational physical activity was not available in our cohort and could represent an additional factor influencing skeletal muscle mass and composition. Future studies should integrate imaging-derived muscle assessment with standardized functional testing. Despite these limitations, our findings provide insights into systemic muscle involvement in bronchiectasis. Larger prospective studies, ideally multicentre, are warranted to confirm these associations and to determine whether nutritional or exercise-based interventions targeting sarcopenia can translate into improvements in clinical outcomes, including exacerbations, infections, and quality of life, in this patient population.

## Conclusions

Reduced muscle density is highly prevalent in patients with bronchiectasis. In addition, body composition impacts significantly on pulmonary function, disease severity, and NTM pulmonary disease risk in this patient population. The use of CT-derived muscle density measurements offers a novel and easily accessible tool for assessing myosteatosis in bronchiectasis, which could improve risk stratification. Future research should focus on further elucidating the mechanisms linking myosteatosis with worse clinical outcomes and exploring targeted interventions to prevent or treat muscle loss in bronchiectasis.

## Supplementary Information

Supplementary Material 1.

## Data Availability

The datasets used and/or analysed during the current study are available from the corresponding author on reasonable request.

## Abbreviations

BACI: Bronchiectasis Aetiology Comorbidity Index
BMI: Body Mass Index
BSI: Bronchiectasis Severity Index
CF: Cystic Fibrosis
COPD: Chronic Obstructive Pulmonary Disease
CT: Computed Tomography
DLCO: Diffusing Capacity of the Lungs for Carbon Monoxide
DXA: Dual-energy X-ray Absorptiometry
E-FACED: Exacerbations–FACED score
FACED: Forced Expiratory Volume in 1 second, Age, Chronic colonization, Extension, Dyspnoea score
FEV₁: Forced Expiratory Volume in 1 second
FVC: Forced Vital Capacity
HRCT: High-Resolution Computed Tomography
HU: Hounsfield Units
Ig: Immunoglobulin
MEF₅₀: Maximal Expiratory Flow at 50% of FVC
MSp: Patients with myosteatosis
NTM: Nontuberculous Mycobacteria
PEF: Peak Expiratory Flow
RV: Residual Volume
TLC: Total Lung Capacity
VMI: Vertebral Muscle Index
wMSp: Patients without myosteatosis
