# Supplementary material for: Quantitative computed tomography body composition analysis for risk stratification in bronchiectasis
Source: Respir Res. 2026 Apr 17;27:236. doi: 10.1186/s12931-026-03670-x (PMC13262219; doi:10.1186/s12931-026-03670-x)
Supplement: Supplementary file 1 — Supplementary Material 1. [file 12931_2026_3670_MOESM1_ESM.docx]

**Quantitative computed tomography body composition analysis for risk stratification in bronchiectasis**

Umberto Semenzato¹*^#^, Virginia Santello¹^#^, Giulia Fichera²^¶^, Daniele Previtero¹^¶^, Chiara Contin^3^, Andrea Rastelli^4^, Rossella Valvason^5^, Marta Zuffellato^6^, Anna Ferrari^7^, Alessandro Micelli², Chiara Giraudo², Andrea Sattin^7^, Anna Maria Cattelan^7^, Paolo Spagnolo¹, Mariaenrica Tinè¹

1 Respiratory Medicine, Department of Cardiac, Thoracic, Vascular Sciences and Public Health, Padova University Hospital - Padua (Italy),

2 Unit of Advanced Clinical and Translational Imaging, Department of Cardiac, Thoracic, Vascular Sciences and Public Health, Padova University, Padova, Italy

3 Respiratory Disease Unit, Ospedale dell'Angelo, Mestre, Venice, Italy.

4 Department of Pneumology, Cittadella Hospital (PD), ULSS6 Euganea, Cittadella, Italy.

5 Pulmonology Unit, Dolo-Mirano Hospital, AULSS3 Serenissima, Venice, Italy.

6 Pulmonology Unit, San Bassiano Hospital, Bassano Del Grappa, Italy

7 Infectious and Tropical Diseases Unit, Padova University Hospital - Padua (Italy)

* corresponding author

^#^Umberto Semenzato and Virginia Santello contributed equally to this work.

^¶^Giulia Fichera and Daniele Previtero contributed equally and shared second authorship.

**Correspondence to**: Umberto Semenzato, Respiratory Medicine, Department of Cardiac, Thoracic, Vascular Sciences and Public Health, Padova University Hospital - 35128 Via Giustiniani 2, Padua (Italy). Email: [umberto.semenzato@aopd.veneto.it](mailto:umberto.semenzato@aopd.veneto.it)

# Supplementary

**Supplementary Results**

Vertebral Muscle Index (VMI) was not significantly correlated with clinical or functional parameters, including age, smoking exposure, exacerbation rate, bronchiectasis severity scores (BSI, FACED, E-FACED, BACI), or lung function. A significant correlation was observed only with BMI (r = 0.274, p = 0.005).

Moreover, there was no correlation between VMI and HU muscle (r = 0.101; p = 0.291).

The BMI was similar among MSp with and without NTM (21.98 [19.99-25.24] vs 22.52 [20.14-24.99]; p = 0.788).

**Supplementary Figures**

**Figure S1**. The aetiology of bronchiectasis in the study population.

Abbreviations: MSp = patients with myosteatosis; wMSp = patients without myosteatosis; ABPA = allergic broncho-pulmonary aspergillosis; COPD = chronic obstructive pulmonary disease; CTD = connective tissue disease; NTM = nontuberculous mycobacteria.

**Figure S2.** Association between FEV_1_ and muscle density.

Scatter plot showing the association between FEV₁ (% predicted) and muscle density. Spearman’s rank correlation coefficient r = 0.23; p = 0.016

Abbreviations: FEV₁= Forced Expiratory Volume in 1 second; HU = Hounsfield Units

**Figure S3.** Association between Bronchiectasis Severity Index and muscle density.

Scatter plot showing the association between Bronchiectasis Severity Index (BSI) and muscle density. Spearman’s rank correlation coefficient r = −0.32; p = 0.012

Abbreviations: BSI = Bronchiectasis Severity Index, HU = Hounsfield Units
